# Assessment of the measurement properties of the Peabody Developmental Motor Scales-2 by applying the COSMIN methodology

**DOI:** 10.1186/s13052-024-01645-6

**Published:** 2024-04-24

**Authors:** Yuanye Zhu, Jiahui Hu, Weibing Ye, Mallikarjuna Korivi, Yongdong Qian

**Affiliations:** Institute of Human Movement and Sports Engineering, College of Physical Education and Health Sciences, Zhejiang Normal University, 321004 Jinhua City, Zhejiang China

**Keywords:** PDMS-2, COSMIN, Motor development, Reliability, Validity, Responsiveness

## Abstract

The Peabody Developmental Motor Scales-2 (PDMS-2) has been used to assess the gross and fine motor skills of children (0–6 years); however, the measurement properties of the PDMS-2 are inconclusive. Here, we aimed to systematically review the measurement properties of PDMS-2, and synthesize the quality of evidence using the Consensus-based Standards for the Selection of Health Measurements Instruments (COSMIN) methodology. Electronic databases, including PubMed, EMBASE, Web of Science, CINAHL and MEDLINE, were searched for relevant studies through January 2023; these studies used PDMS-2. The methodological quality of each study was assessed by the COSMIN risk-of-bias checklist, and the measurement properties of PDMS-2 were evaluated by the COSMIN quality criteria. Modified GRADE was used to evaluate the quality of the evidence. We included a total of 22 articles in the assessment. Among the assessed measurement properties, the content validity of PDMS-2 was found to be sufficient with moderate-quality evidence. The structural validity, internal consistency, test-retest reliability and interrater reliability of the PDMS-2 were sufficient for high-quality evidence, while the intrarater reliability was sufficient for moderate-quality evidence. Sufficient high-quality evidence was also found for the measurement error of PDMS-2. The overall construct validity of the PDMS-2 was sufficient but showed inconsistent quality of evidence. The responsiveness of PDMS-2 appears to be sufficient with low-quality evidence. Our findings demonstrate that the PDMS-2 has sufficient content validity, structural validity, internal consistency, reliability and measurement error with moderate to high-quality evidence. Therefore, PDMS-2 is graded as ‘A’ and can be used in motor development research and clinical settings.

**Supplementary Information:**

The online version contains supplementary material available at 10.1186/s13052-024-01645-6.

## Introduction

Motor development refers to the ability of children to move and interact with the environment and is very important in early childhood [1]. Proper motor development provides an opportunity for children to explore and participate in the world around them [2]. Several studies have shown that motor development is closely associated with children’s cognitive ability [3], language [4], executive functioning [5], and quality of life [6]. Children with poor motor development reportedly have poor academic performance as well as depression and anxiety [7]. In addition, impaired motor development in early childhood can impact learning abilities, which may persist through adolescence or even later in life [8]. Motor disorders in children are associated with a lower quality of life in several domains, including physical, cognitive, emotional and social functioning [6]. Children with motor dyspraxia (developmental disorder) require motor intervention to promote their motor skills and to prevent postural abnormalities [9]. Therefore, early prediction of motor function is important for further intervention and education [10]. Many assessment instruments or scales have been developed to accurately and efficiently screen for motor development problems in children [11, 12]. The Peabody Developmental Motor Scales-2 (PDMS-2) is widely used in paediatric practice and research studies to assess the gross and fine motor skills of children from birth to 6 years of age [13]. The PDMS-2 has been improved and updated based on reviews of the PDMS, comments and queries from the testers and the authors’ own experiences [14]. The key changes in PDMS include the collection of a more representative sample, the introduction of a different test structure and more specific scoring criteria [15].

The measurement properties of an instrument were described and defined by the COnsensus-based Standards for the selection of health Measurements INstruments (COSMIN). According to the COSMIN methodology, reliability, validity and responsiveness are the main domains. The reliability was categorized into test-retest, interrater and intrarater reliability, and validity was categorized into content, construct (structural, cross-cultural, hypothesis testing) and criterion validity [16]. Since the publication of PDMS-2, many studies have examined the measurement properties of this scale. The measurement properties of the original version have been assessed by English-speaking countries [17–19], while the measurement properties of the translated versions have been assessed by non-English-speaking countries [20, 21]. Although several studies have confirmed the reliability and validity of the PDMS-2 device to be sufficient, there are some contradictory reports on its reliability and validity. For example, the concurrent validity of the PDMS-2 and the Bayley Scales of Infant Development II Motor Scale (BSID-II) was simultaneously reported to be “high correlation” [22] and “low correlation” [19]. Despite the heterogeneity of studies on the measurement properties of PDMS-2, no systematic review has addressed this issue. Since PDMS-2 is widely used by clinicians, therapists, psychologists and diagnosticians [14], establishing consistent evidence on its measurement properties is highly warranted.

The COSMIN methodology is typically employed to evaluate the measurement properties of various tools/scales of a certain field [23, 24]. Hulteen et al. employed the COSMIN methodology in their systematic review of the measurement properties of several motor assessment scales in children and adolescents [25]. The COSMN methodology can also be used to review the measurement properties of a single measurement instrument, such as the Body Image Scale [26]. As reported results are inclusive of the measurement properties (reliability, validity, and responsiveness) of PDMS-2, the COSMIN could be an alternative methodology to delineate this inconsistency. Therefore, we searched for studies that determined the measurement properties of PDMS-2 and employed the COSMIN methodology to conduct a systematic review of the measurement properties of PDMS-2. In this review, we summarize the state of research on the measurement properties of PDMS-2 and synthesize the quality of evidence via the COSMIN methodology.

## Methods

### Literature search strategy

The PubMed, EMBASE, Web of Science, CINAHL and MEDLINE databases were searched for relevant studies that assessed the different measurement properties of PDMS-2 through January 2023. The search terms or keywords used to identify the name of the scale/instrument (PDMS-2) were “Peabody developmental motor scales-2” OR “PDMS-2” OR “Peabody developmental motor scales-second edition” OR “Peabody developmental motor scales-2nd “. The search term utilized to determine the scale measurement properties was a filter developed by the Patient Reported Outcome Measures (PROMs) Group at the University of Oxford (a high-sensitivity search filter that has been validated by Terwee et al. [27]. For the article search, we followed the latest version of the Preferred Reporting Items for Systematic Reviews and Meta-Analyses (PRISMA, 2020) guidelines [28]. The full texts of the selected articles were downloaded from the journal’s homepage. In addition, we contacted our university library or external collaborators for the full-text articles upon necessary. The study protocol was registered in PROSPERO (https://www.crd.york.ac.uk/prospero/; CRD42022376335).

### Inclusion and exclusion criteria

The included literature met the following criteria: (1) the study was conducted on children aged 0–6 years; (2) the study addressed the evaluation of the PDMS-2 measurement properties; and (3) at least one of the scale’s measurement properties was evaluated in the study. The measurement properties of the PDMS-2 include content validity, structural validity, internal consistency, cross-cultural validity/measurement invariance, reliability, measurement error, criterion validity, hypothesis testing for construct validity, and responsiveness. The collected literature was excluded if it met any of the following criteria: (1) used PDMS-2 to investigate children’s motor development; (2) used PDMS-2 to assess the effectiveness of an intervention; (3) was a review and systematic review; or (4) had only an abstract without a full-text article or nonpeer review.

### Literature selection and data extraction

The literature search, article selection and data extraction were independently performed by two researchers (YZ and JH), and the results were compared with the help of another author (YQ). Any disagreements were resolved by discussion with other review authors (WY and MK). The literature was imported into EndNote, and duplicates were first excluded. Subsequently, the titles and abstracts of the collected articles were read, and irrelevant articles were excluded. The full texts of the remaining articles were subsequently read and screened according to our study criteria.

The following information was extracted from the literature: first author name, year of publication, studied population and source, region, sample size, age and sex of the children, use of the PDMS-2 language, measurement properties of the PDMS-2 (content validity, structural validity, internal consistency, cross-cultural validity/measurement invariance, reliability, measurement error, criterion validity, hypothesis testing for construct validity, and responsiveness), and data on the measurement properties.

### Evaluation of the risk of bias and quality of evidence of the included studies

We used the COSMIN risk of bias checklist [29] to assess the methodological quality of the studies. The checklist consists of ten sections, including “PROM development, content validity, structural validity, internal consistency, cross-cultural validity/measurement invariance, reliability, measurement error, criterion validity, hypothesis testing for construct validity, and responsiveness”. Appropriate boxes were selected according to the measurement properties of the study. The methodological quality of the studies was assessed as “very good”, “adequate”, “doubtful” or “inadequate” on an item-by-item basis according to the standard score given in the boxes. The overall methodological quality rating of the studies was based on the “worst score principle”. The worst score of the criteria in the box was regarded as the overall methodological quality rating of the study.

The quality of evidence was synthesized according to the modified version of the Grading of Recommendations Assessment, Development and Evaluation (GRADE) method [24]. This method is an improvement on the original version to accommodate the COSMIN method. The evidence levels could be categorized as “high”, “moderate”, “low” or “very low” according to the standard. The starting level of evidence for the included studies was “high”, and the data were subsequently downgraded according to the characteristics of the included studies. Unlike the original GRADE method, the modified version removes the “publication bias” factor. The quality of evidence was downgraded according to the risk of bias, inconsistency, indirectness, and imprecision.

### Overall rating of the measurement properties

The overall rating of each measurement property of the PDMS-2 was assessed by the COSMIN methodology for systematic reviews of the PROM user manual (COSMIN manual) [30] and the COSMIN methodology for assessing the content validity of the PROM user manual [31]. The items included “content validity, structural validity, internal consistency, cross-cultural validity/measurement invariance, reliability, measurement error, criterion validity, hypothesis testing for construct validity, and responsiveness” (Table S1). The reported items for each measurement property were rated as “sufficient (+), “insufficient (-), or “indeterminate (?)” (Table S2). The overall rating of each measurement property was given as “sufficient (+)”, “insufficient (-)”, “inconsistent (±)”, or “indeterminate (?)”. Inconsistent results were analysed in groups to explore the reasons for this difference.

For reliability, studies were considered sufficient if the Pearson correlation coefficient [32] or Spearman’s rho correlation coefficient [33] was ≥ 0.80. Hypothesis testing for construct validity requires the reviewer team to set hypotheses in advance. The hypothesis for this study was as follows: for construct convergent or concurrent validity, the correlation coefficient was expected to be ≥ 0.50 for the correlations with the comparator instrument if a similar construct was measured with respect to the PDMS-2. Construct validity was rated as sufficient (+) if at least 75% of the results were in accordance with the hypotheses, insufficient (−) if at least 75% of the results were not, or indeterminate (?) if no hypotheses were defined.

## Results

### Literature search results

From our database search, we identified a total of 529 articles, including 95 articles from PubMed, 103 from EMBASE, 156 from Web of Science, 48 from CINAHL, and 127 from MEDLINE. The search was performed until January 31, 2023, without restriction of early publication time.

**Fig. 1:**
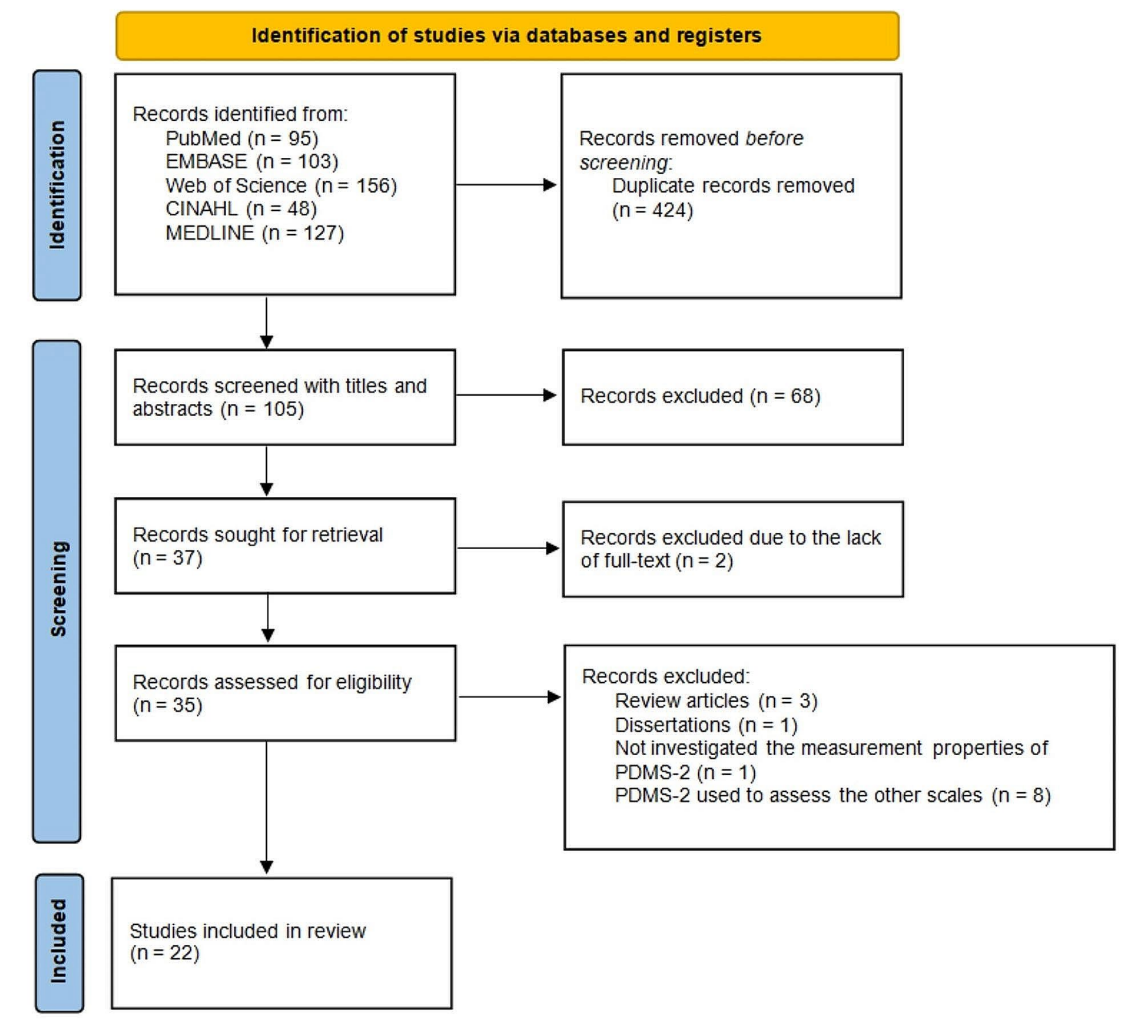
Flow diagram of the article selection according to the PRISMA

All identified articles were imported to EndNote, and 424 duplicates were removed. The titles and abstracts of the remaining 105 articles were screened, and 68 irrelevant articles were excluded, resulting in 37 additional articles. Then, two articles were excluded due to unavailability of the full text (conference abstracts), and 35 were assessed for eligibility. We further excluded 13 articles for the following reasons: three articles were reviews [15, 34, 35], one was a dissertation [36], one study did not investigate the measurement properties of PDMS-2 [37], and eight studies used PDMS-2 to assess other scales [2, 38–44]. Finally, 22 articles were included in our assessment. The detailed selection process and number of articles in each step are shown in Fig. 1.

### Characteristics of the included studies

The characteristics of the included articles are shown in Table 1. The studies were intercontinental, mainly from Europe [21, 45–50], followed by Asia [20, 22, 51–55] and North America [14, 17–19, 56, 57]. Specifically, six studies were from the USA [14, 17–19, 56, 57]; four studies were from Taiwan, China [51–54]; three, Portugal [21, 45, 46]; two, Brazil [58, 59]; two, South Korea [20, 55]; one, Belgium [47]; Spain [50]; the Netherlands [48]; Iran [22]; and the UK [49]. The participants in these studies were both normal [14, 19–21, 45, 46] and exceptional [17, 18, 22, 47–59] children. Exceptional children were identified as having various disabilities, such as developmental delays [17, 47, 48, 51, 52, 56, 57], premature birth [49, 58, 59] and neurological diseases [18, 50, 53–55]. The age of the children ranged from 0 to 6 years.

**Table 1:** Basic characteristics of the included articles

Authors (year)	Population characteristics				Research characteristics of PDMS-2		
	N	Age	Sex (M/F)	Studied population	Country/Region	PDMS-2 language	Measurement properties
Álvarez Gonzalo et al. (2021) [50]	74	0–36 m	NR	Children with neurodevelopmental disorders	Spain	Spanish	Internal consistency, Inter-rater reliability
Chien & Bond (2009) [52]	419	0–60 m	238/181	Normal children and fine motor delays children	China Taiwan	Chinese	Unidimensional
Connolly et al. (2006) [19]	15	12 m	9/6	Normal children	America	English	Concurrent validity
Connolly et al. (2012) [56]	48	1–26 m	32/16	Developmentally delayed children	America	English	Concurrent validity
Folio & Fewell (2000) [14]	567	0–11 m	284/273	Normal children	America	English	Structure validity, Test-retest reliability, Inter-rater reliability
Gill et al. (2019) [49]	184	18 m	95/89	Premature children	England	English	Concurrent validity
Holloway et al. (2019) [18]	22	48–71 m	21/1	Children with autism spectrum disorder	America	English	Concurrent validity
Kim et al. (2021) [55]	84	24–71 m	50/34	Typical children and Cerebral Palsy children	Korea	Korean	Test-retest reliability
Lee et al. (2019) [20]	50	42–71 m	25/25	Normal children	Korea	Korean	Inter-rater reliability, Concurrent validity
Lin et al. (2020) [51]	223	13–36 m	157/66	Developmentally delayed children	China Taiwan	Chinese	Concurrent validity
Maring & Elbaum (2007) [17]	30	12–44 m	18/12	Developmentally delayed children	America	English	Concurrent validity
Provost et al. (2004) [57]	110	3–41 m	75/35	Developmentally delayed children	America	English	Concurrent validity
Rebelo et al. (2021) [45]	392	12–48 m	199/193	Normal children	Portugal	Portuguese	Internal consistency, Test-retest reliability, Structure validity
Saraiva et al. (2011) [21]	540	36–71 m	255/285	Normal children	Portugal	Portuguese	Internal consistency, Structure validity
Saraiva et al. (2013) [46]	540	36–71 m	255/285	Normal children	Portugal	Portuguese	Cross-cultural validity
Tavasoli et al. (2014) [22]	88	18 ± 2 m	44/44	58 low and 30 normal weight children	Iran	Iranian	Test-retest reliability, Convergent validity
Valentini & Zanella (2022) [58]	637	0–71 m	312/325	Premature and normal children	Brazil	Portuguese	Unidimensional
van Hartingsveldt et al. (2005) [48]	36	4–5 y	32/4	18 children with and 18 without minor motor development problems	Netherlands	Dutch	Test-retest reliability, Inter-rater reliability, Convergent validity
Waelvelde et al. (2007) [47]	31	4–5 y	27/4	Developmentally delayed children	Belgium	English	Convergent validity
Wang et al. (2006) [53]	32	27–64 m	23/9	Cerebral palsy children	China Taiwan	English	Test-retest reliability, Responsiveness
Wuang et al. (2012) [54]	141	36–72 m	52/89	The children with an adaptive disability	China Taiwan	English	Internal consistency, Test-retest reliability, Concurrent validity, Responsiveness, Measurement error
Zanella et al. (2021) [59]	637	0–71 m	312/325	Premature and normal children	Brazil	Portuguese	Content validity, Structure validity, Test-retest reliability, Inter-rater reliability, Intra-rater reliability

Note: M/F = male/female, y = years, m = month, NR = Not Report

### Synthesis of evidence for the measurement properties of PDMS-2

The overall assessment of the PDMS-2 measurement properties and the corresponding quality of evidence for each measurement property are shown in Table 2. The detailed quality of evidence data are provided in the supplementary material (Table S3).

**Table 2:** Summary of the findings

Measurement property	Summary or pooled results	Overall rating	Quality of evidence
**Content validity**	**Content validity**: [59]	**Qualitative summary: Sufficient (+)**	**Moderate: study bias**
	Relevance	Sufficient (+)	
	Comprehensiveness	Sufficient (+)	
**Structural validity**	**Structural validity**: [14, 21, 45, 59]	**Qualitative summary: Sufficient (+)**	**High: all studies are very good**
	TLI: 0.98-1.00 [14, 21, 45]	Sufficient (+)	
	CFI: 1.0 [59]	Sufficient (+)	
	Total sample size: 2136		
**Internal consistency**	**Internal consistency**: [21, 45, 50, 54]	**Qualitative summary: Sufficient (+)**	**High: all studies are very good**
	Reflex: α 0.999	Sufficient (+)	
	Stationary: α 0.86–0.999	Sufficient (+)	
	Locomotion: α 0.89–0.999	Sufficient (+)	
	Manipulation: α 0.87–0.991	Sufficient (+)	
	Grasping: α 0.76–0.999	Sufficient (+)	
	Visual–Motor: α 0.89–0.999	Sufficient (+)	
	Total sample size: 1147		
**Reliability**	**Reliability**: [14, 20, 22, 45, 48, 50, 53–55, 59]	**Qualitative summary: Sufficient (+)**	**High: all studies are very good**
	**Test–retest reliability**: [14, 22, 45, 48, 53–55, 59]	**Qualitative summary: Sufficient (+)**	**High: all studies are very good**
	GMQ: ICC 0.75–0.99 [22, 45, 53, 54]	Sufficient (+)	
	FMQ: ICC 0.71–0.99 [22, 45, 53, 54]	Sufficient (+)	
	GMQ: *r* 0.84–0.99 [14, 55, 59]	Sufficient (+)	
	FMQ: *r* 0.73–0.99 [14, 55, 59]	Sufficient (+)	
	FMQ: *ρ* 0.84–0.98 [48]	Sufficient (+)	
	Total sample size: 1360		
	**Inter-rater reliability**: [14, 20, 48, 50, 59]	**Qualitative summary: Sufficient (+)**	**High: all studies are very good**
	Reflex: ICC 0.758–0.920 [50, 59]	Sufficient (+)	
	Stationary: ICC 0.985–0.999 [20, 50, 59]	Sufficient (+)	
	Locomotion: ICC 0.990-1.000 [20, 50, 59]	Sufficient (+)	
	Manipulation: ICC 0.972–0.999 [20, 50, 59]	Sufficient (+)	
	Grasping: ICC 0.941–0.991 [20, 50, 59]	Sufficient (+)	
	Visual-motor: ICC 0.988-1.000 [20, 50, 59]	Sufficient (+)	
	GMQ: *r* 0.97 [14]	Sufficient (+)	
	FMQ: *r* 0.98 [14]	Sufficient (+)	
	TMQ: *r* 0.96 [14]	Sufficient (+)	
	FMQ: *ρ* 0.94–0.99 [48]	Sufficient (+)	
	Total sample size: 367		
	**Intra-rater reliability**: [59]	**Qualitative summary: Sufficient (+)**	**Moderate: imprecision due to small sample size**
	Reflex: ICC 0.96	Sufficient (+)	
	Stationary: ICC 0.95–0.97	Sufficient (+)	
	Locomotion: ICC 0.98–0.99	Sufficient (+)	
	Manipulation: ICC 0.98	Sufficient (+)	
	Grasping: ICC 0.92–0.94	Sufficient (+)	
	Visual-motor: ICC 0.92–0.98	Sufficient (+)	
	Total sample size: 80		
**Measurement error**	**Measurement error**: [54]	**Qualitative summary: Sufficient (+)**	**High: one study is very good**
	SDC (MDC): 7.76 < MIC (MCID): 8.39	Sufficient (+)	
	Total sample size: 191		
**Hypothesis testing for construct** **validity**	**Construct validity**: [17–20, 22, 47–49, 51, 54, 56, 57]	**Qualitative summary: Sufficient (+)**	**Moderate: inconsistent results**
	**PDMS-GM-2 and EIDP** **Concurrent validity**: [17]	**Qualitative summary: Sufficient (+)**	**Low: severe imprecision due to small sample size**
	GMQ: *r* 0.91	Sufficient (+)	
	Total sample size: 30		
	**PDMS-GM-2 and M-FUN** **Concurrent validity**: [18]	**Qualitative summary: Sufficient (+)**	**Low: severe imprecision due to small sample size**
	GMQ: *r* 0.851	Sufficient (+)	
	Total sample size: 22		
	**PDMS-2 and Bayley-III** **Concurrent validity**: [49, 51, 56]	**Qualitative summary: Sufficient (+)**	**High: all studies are very good**
	GMQ: *r* 0.59–0.90 [49, 56]	Sufficient (+)	
	FMQ: *r* 0.50–0.94 [49, 51, 56]	Sufficient (+)	
	TMQ: *r* 0.69–0.95 [49, 56]	Sufficient (+)	
	Total sample size: 455		
	**PDMS-2 and BSID-II** **Convergent validity**: [22] **Concurrent validity**: [19, 57]	**Qualitative summary**: **Sufficient (+)**	**High: most studies are very good**
	NC GMQ: *r* 0.30 [19]	Insufficient (-)	Low: imprecision due to small sample size
	NC FMQ: *r* 0.22 [19]	Insufficient (-)	
	NC sample size: 15		
	EC GMQ: *r* 0.75–0.93 [22, 57]	Sufficient (+)	High: two studies are very good
	EC FMQ: *r* 0.67–0.91 [22, 57]	Sufficient (+)	
	EC sample size: 198		
	Total sample size: 213		
	**PDMS-2 and BOT-2** **Concurrent validity**: [20, 54]	**Qualitative summary: Sufficient (+)**	**High: all studies are very good**
	All sub-scales: *r* 0.651–0.951 [20]	Sufficient (+)	
	All sub-scales: *ρ* 0.84–0.88 [54]	Sufficient (+)	
	Total sample size: 687		
	**PDMS-2 and M-ABC** **Convergent validity**: [47, 48]	**Qualitative summary**: **Inconsistent (±)**	**Very low: inconsistent results and small sample size**
	GMQ: *r* 0.71 [47]	Sufficient (+)	
	FMQ: *r* 0.48–0.69 [47, 48]	Inconsistent (±)	
	Total sample size: 67		
**Responsiveness**	**Before and after intervention**: [53, 54]	**Qualitative summary: Sufficient (+)**	**Low: severe bias**
	ES = 0.47–0.74; SRM = 0.35–0.70 [54]	Sufficient (+)	
	Sample size: 141		
	GRI-R range: 1.7–2.3 [53]	Sufficient (+)	
	Sample size: 32		

Note: ICC = Intraclass Correlation Coefficient, GMQ = Gross Motor Quotient, FMQ = Fine Motor Quotient, TMQ = Total Motor Quotient, MIC (MCID) = Minimal Important Change, SDC (MDC) = Smallest Detectable Change, PDMS-GM-2 = PDMS-2 Gross Motor scale, EIDP = Early Intervention Developmental Profile, M-FUN = Miller Function and Participation Scales, Bayley-III = the Bayley Scales of Infant and Toddler Development, 3rd edition, BSID-II = the Bayley Scales of Infant Development II Motor Scale, BOT-2 = Bruininks-Oseretsky Test of Motor Proficiency-Second Edition, M-ABC = Movement Assessment Battery for Children, ES = Effect Size, SRM = Standardised Response Mean, GRI-R = Guyatt’s responsiveness Ratio for Responsiveness, *ρ* = Spearman’s rho correlation coefficient, *r* = Pearson’s correlation coefficients, TLI = Tucker-Lewis index, CFI = Comparative Fit Index,α = Cronbach’s alpha value, All sub-scales = Reflex, Stationary, Locomotion, Manipulation, Grasping, Visual-motor sub-scales NC = Normal Children, EC = Exceptional Children

#### Content validity

Of the 22 included articles, only one study methodologically assessed the content validity of the PDMS-2 standard recommended by the COSMIN [59]. The study systematically assessed the content validity of the PDMS-2 by interviewing experts in the field and judged the relevance and comprehensiveness of the scale. The overall rating of the results for content validity was found to be sufficient, and the quality of evidence was moderate. Since this study did not report comprehensibility, it was not possible to judge the overall rating of comprehensibility (Table 2).

#### Structural validity

Four of the 22 included articles assessed the bifactor structural validity of the PDMS-2 by classical test theory (CTT) [14, 21, 45, 59]. The overall rating of the results for structural validity was found to be sufficient. The quality of evidence was high, and all studies were judged as very good (Table 2).

#### Internal consistency

Two studies examined the unidimensionality of the PDMS-2 subscales through Rasch analysis and indicated that most items on the scale met the unidimensionality requirement [52, 58]. Four of the 22 included articles assessed the internal consistency of the PDMS-2 [21, 45, 50, 54]. The Cronbach’s alpha values for the internal consistency of PDMS-2 were 0.999 (Reflex), 0.86–0.999 (Stationary), 0.89–0.999 (Locomotion), 0.87–0.991 (Manipulation), 0.76–0.999 (Grasping) and 0.89–0.999 (Visual–Motor). The overall rating was sufficient, and the quality of evidence of all included studies was high for internal consistency (Table 2).

#### Cross-cultural validity/measurement invariance

Of the 22 included articles, only one study assessed the cross-cultural validity of the PDMS-2 [46]. However, the methodology used in this study did not meet the COSMIN methodological requirements.

#### Reliability

Ten studies assessed the reliability of the PDMS-2 [14, 20, 22, 45, 48, 50, 53–55, 59]. According to the COSMIN manual [30], these studies can be divided into test-retest reliability, interrater reliability and intrarater reliability.

Eight studies assessed the test-retest reliability of the PDMS-2 [14, 22, 45, 48, 53–55, 59]. These studies mainly used the intraclass correlation coefficient (ICC) [22, 45, 53, 54], Pearson correlation coefficient (r) [14, 55, 59] and Spearman’s rho correlation coefficient (ρ) [48] to judge test-retest reliability. The ICCs for the test-retest reliability of the PDMS-2 were 0.75–0.99 (gross motor subscale [GMS]) and 0.71–0.99 (fine motor subscale [FMS]). The Pearson correlation coefficients were 0.84–0.99 (GMS) and 0.73–0.99 (FMS); the Spearman’s rho correlation coefficients were 0.84–0.98 (FMS). The overall rating of the results for test-retest reliability was found to be sufficient, and the quality of evidence was high (Table 2).

As shown in Table 2, five studies assessed the interrater reliability of the PDMS-2 [14, 20, 48, 50, 59]. These studies mainly used the ICC [20, 50, 59], Pearson correlation coefficient [14] and Spearman’s rho correlation coefficient [48] to judge interrater reliability. The ICCs for the interrater reliability of the PDMS-2 were 0.758–0.920 (Reflex), 0.985–0.999 (Stationary), 0.990-1.000 (Locomotion), 0.972–0.999 (Manipulation), 0.941–0.991 (Grasping) and 0.988-1.000 (Visual-motor); the Pearson correlation coefficient was 0.97 (GMS), 0.98 (FMS) and 0.96 (Total Motor scale); and the Spearman’s rho correlation coefficients were 0.94–0.99 (FMS). The overall rating of the results for the interrater reliability was found to be sufficient. The quality of evidence of the studies was judged to be high, and all studies were identified as very good.

One study assessed the intrarater reliability of the PDMS-2 [59]. The ICC for the intrarater reliability of the PDMS-2 was more than 0.70. However, due to the imprecision of the included studies (total sample size 80, i.e., < 100), the quality of evidence was graded as moderate. Therefore, there was sufficient moderate-quality evidence for the intrarater reliability of the PDMS-2 (Table 2). Taken together, the high-quality evidence from our assessment demonstrated that the reliability of the PDMS-2 was sufficient.

#### Measurement error

One study evaluated the measurement error of PDMS-2 [54]. The smallest detectable change (SDC) was 7.76, and the minimal important change (MIC) was 8.39, which met the criterion of sufficient survival (+, SDC < MIC). The quality of evidence was high. Therefore, there was sufficient high-quality evidence for the measurement error of PDMS-2 (Table 2).

#### Hypothesis testing for construct validity

There is no ‘gold standard’ in the field of children’s motor development assessment. Therefore, concurrent validity as a part of criterion validity is classified as evidence of construct validity recommended by the COSMIN [30].

A total of 13 studies evaluated the construct validity of the PDMS-2 [17–20, 22, 47–49, 51, 54, 56, 57]. These studies assessed the construct validity of the PDMS-2 by examining the correlation of the PDMS-2 with similar domain measurement instruments. These measurement instruments included the Early Intervention Developmental Profile (EIDP) [17], Miller Function and Participation Scales (M-FUN) [18], the Bayley Scales of Infant and Toddler Development, 3rd edition (Bayley-III) [49, 51, 56], the Bayley Scales of Infant Development-II (BSID-II) Motor Scale [19, 22, 57], the Bruininks-Oseretsky Test of Motor Proficiency-Second Edition (BOT-2) [20, 54] and the Movement Assessment Battery for Children (M-ABC) [47, 48] (Table 2).

One study assessed the concurrent validity of the PDMS-2 Gross Motor scale (PDMS-GM-2) with the EIDP [17]. The overall rating results showed that the concurrent validity was sufficient. Because the sample size (30 children) was less than 50, the quality of evidence was low. Overall, there was sufficient low-quality evidence for the concurrent validity of the PDMS-GM-2 with the EIDP. One study assessed the concurrent validity of the PDMS-GM-2 with the M-FUN [18]. The overall rating results showed that the concurrent validity was sufficient, but the quality of evidence was low due to the small sample size (22 children, i.e., < 50). Overall, our results showed that there was sufficient low-quality evidence for the concurrent validity of the PDMS-GM-2 with M-FUN (Table 2).

Three studies assessed the concurrent validity of the PDMS-2 with the Bayley-III [49, 51, 56]. The overall rating of the results for the concurrent validity of the PDMS-2 with the Bayley-III was found to be sufficient, and the quality of the evidence was high. Three studies assessed the concurrent [19, 57] and convergent [22] validity of the PDMS-2 with the BSID-II. Of these three studies, two involved the recruitment of exceptional children [22, 57]; the overall rating was judged as sufficient (+), and the quality of evidence was high. One study recruited normally developing children [19]; the overall rating was judged as insufficient (-), and the quality of evidence was low. Our assessment revealed that the results of the PDMS-2 device with BSID-II appeared to be sufficient, and the quality of evidence was high (Table 2).

Two studies assessed the concurrent validity of the PDMS-2 with the BOT-2 [20, 54]. The overall rating of the results for the concurrent validity of the PDMS-2 with the BOT-2 was found to be sufficient, and the quality of evidence was high. Furthermore, two studies [47, 48] examined the convergent validity of PDMS-2 with M-ABC. These two studies met the requirement of correlation in PDMS-GM-2 but not in PDMS-FM-2. Therefore, the convergent validity of PDMS-2 with M-ABC was inconsistent. The quality of evidence was very low due to the small sample size (67 children, < 100) and inconsistent results. Thus, there is inconsistent very low-quality evidence for the convergent validity of PDMS-2 with M-ABC (Table 2).

#### Responsiveness

Two studies assessed the responsiveness of PDMS-2 [53, 54]. The overall rating of the results was sufficient. However, the quality of evidence was low because the study was severely biased according to the COSMIN risk of bias assessment checklist [29]. These results indicate that even low-quality evidence showed sufficient responsiveness of PDMS-2 (Table 2).

## Discussion

To the best of our knowledge, this is the first systematic review in which the COSMIN methodology was used to assess the measurement properties of PDMS-2. In this study, we evaluated the different properties of PDMS-2, which were reported in 22 articles. According to the COSMIN manual, any measurement instrument or scale with sufficient evidence for content validity (any level quality) or internal consistency (at least low quality) can be categorized as “A” [30]. Our results showed that the content validity of the PDMS-2 had sufficient moderate-quality evidence, and the internal consistency of the PDMS-2 had sufficient high-quality evidence. These findings revealed that PDMS-2 can be graded as ‘A’, which can be used in motor development research and in clinical settings. The COSMIN manual further states that the results obtained from any “A” grade scale can be trusted [30].

According to the COSMIN manual, content validity is the most important property of a measurement instrument or scale [30]. Bums and Grove stated that content validity is obtained from three sources: literature, patient judgement (judgement of representatives of the relevant populations), and expert judgement [60]. The most commonly used source of content validity is expert judgement [61], and the COSMIN method combines patient judgement with expert judgement to assess three parts of content validity: relevance, comprehensiveness, and comprehensibility [30]. In our assessment, only one study reported the content validity of the PDMS-2 [59]. However, in this study we examined the content validity of the PDMS-2 by asking experts in related fields but not patients/participants [59]. When using the PDMS-2, patients (children) must complete their movements only following the instructions of the evaluator and do not need to understand the meaning of the PDMS-2 items [14]. Therefore, no studies assessing the comprehensibility of PDMS-2 were found, but we still consider the content validity of PDMS-2 to be sufficient.

For the assessment of structural validity, the COSMIN quality criterion includes two criteria, namely, CTT and item response theory (IRT) [30, 62]. All the studies addressing structural validity in our analyses used the CTT method. Although the CTT easily assesses structural validity, the results from the IRT are said to be more reliable in educational and psychometric fields [63]. Due to its high accuracy, IRT is a highly validated method for assessing the structural validity of PDMS-2 [63]. However, at present, no study has used the IRT to evaluate the structural validity of the PDMS-2, and further studies are necessary to address the importance of IRT.

According to the COSMIN manual, cross-cultural validity/measurement invariance has been defined as “the degree to which the performance of the items on a translated or culturally adapted measurement instruments are an adequate reflection of the performance of the items of the original version of the measurement instruments” [30]. In our analyses, we determined that no studies have assessed the cross-cultural validity/measurement invariance of the PDMS-2 by the COSMIN recommended method. We suggest further research on the cross-cultural validity/measurement invariance of the PDMS-2.

The results of the construct validity test demonstrated that the PDMS-2 is well correlated with most of the same-domain measurement instruments. However, the results of the three studies of the PDMS-2 device with BSID-II differed, which might be due to differences in sample type. Of these three studies, one study recruited normally developing children [19], and two studies recruited exceptional children [22, 57]. The concurrent validity of the PDMS-2 with the BSID-II among normal children was insufficient because of the small sample size (*n* = 15, i.e., < 50) [19]. However, the concurrent or convergent validity among exceptional children was found to be sufficient for obtaining high-quality evidence (sample size 198, > 100) [22, 57]. The COSMIN stated that high-quality studies provide stronger evidence than low-quality studies and can be considered decisive in determining the overall rating when ratings are inconsistent [30]. Overall, our findings revealed that the results of the assessment of PDMS-2 with BSID-II were sufficient. Next, we addressed the convergent validity of the PDMS-2 and M-ABC devices in two studies [47, 48]; the results were sufficient for the gross motor quotient (GMQ) and inconsistent for the fine motor quotient (FMQ). As the sample size was small and the assessment ratings were inconsistent, the quality of PDMS-2 and M-ABC was considered very low evidence.

The risk of bias of reliability and measurement error was not judged according to the retest interval recommended by the COSMIN risk of bias checklist (approximately two weeks) due to the rapid growth rate of children aged 0 to 6 years. However, we judged the risk of bias in the studies (approximately one week) using another method described by Lee et al. [32]. A suitable measurement error requires that the smallest detectable change (SDC) in the measurement instrument is less than the MIC [64]. Only one study was conducted on the SDC and MIC [54]. The MIC is the best result that can be calculated from multiple studies and using multiple anchors [65]. Therefore, it is clear that one study alone is not convincing and involves multiple anchors, and we suggest further studies to verify the MIC results.

Responsiveness measures the ability of a scale to change over time in the construct to be measured [30]. The results of the two included studies [53, 54] showed sufficient responsiveness of PDMS-2, but the quality of evidence of these two studies was low. There are two reasons for these results. First, these two studies did not describe the intervention details. The second reason is that Wang et al. [53] used a statistical method (Guyatt’s responsiveness ratio), which is not recommended by COSMIN [30]. According to the COSMIN manual, Guyatt’s responsiveness ratio takes the minimal important change into account [30]. A marginally important change concerns the interpretation of the change score, not the validity of the change score [30]. Low-quality evidence does not mean validating the sufficient or insufficient responsiveness of the PDMS-2 before and after the intervention.

In addition to the abovementioned outcome measures in COSMIN, interpretability and feasibility are also important variables for evaluating the measurement properties of PDMS-2 [30]. In our assessment, one study [54] reported no ceiling or floor effects when using the PDMS-2 to assess the motor development of children. Reporting such no ceiling or floor effects indicates good interpretability of the PDMS-2. According to the results of previous studies of PDMS-2 [14], we assumed that the use of PDMS-2 is highly feasible and that a specific environment and/or equipment are not necessary to assess motor development in children.

The synthesized evidence of the measurement properties of PDMS-2 is comparable to that of other well-known similar domain measurement instruments, such as M-ABC, BOT-2, Bayley-III, and BSID-II. For instance, a previous study reported that the interrater reliability, test-retest reliability and content validity of the M-ABC were good, but mixed results were reported for internal consistency and cross-cultural validity [66]. The BOT-2 scale was reported to have excellent interrater reliability, test-retest reliability, and internal consistency [66]. Another study reported that the internal consistency and test-retest reliability of the Bayley-III were good [35]. In addition, the interrater reliability, internal consistency, and test-retest reliability of the BSID-II were reported to be sufficient [67]. Our findings demonstrate that the PDMS-2 has sufficient content validity, structural validity, internal consistency, reliability and measurement error with moderate to high-quality evidence.

### Limitations and future perspectives

Our results could not establish the quality of evidence for the cross-cultural validity of PDMS-2 because few or no studies have assessed the cross-cultural validity of PDMS-2 via the COSMIN-recommended methodology. For the article search, the Cochrane reviews used various additional sources, including dissertations, editorials, and conference proceedings. However, the probability of finding additional relevant articles for systematic reviews from these sources appears to be low [24]. As we excluded the nonpeer reviewed articles in our study, our conclusions may not be influenced by these articles; however, we cannot completely exclude them.

To date, no study has addressed the cross-cultural validity of PDMS-2 by the COSMIN recommended method. In addition, only one study assessed the measurement error of PDMS-2. Therefore, further studies are necessary to assess the cross-cultural validity and measurement error of PDMS-2. These measurement properties can be used in the assessment to determine the overall rating and quality of evidence by the COSMIN methodology. We further suggest that future studies on the responsiveness of PDMS-2 that can be used in the COSMIN methodology.

## Conclusions

Assessment results from the COSMIN methodology showed that the PDMS-2 has sufficient high-quality evidence for structural validity and internal consistency. The reliability and measurement error of the PDMS-2 also demonstrated sufficient high-quality evidence. However, no adequate or low-quality evidence was found for the cross-cultural validity/measurement invariance and responsiveness of the PDMS-2. On the other hand, very low-quality evidence for convergent validity suggested that the PDMS-FM-2 was inconsistently correlated with the M-ABC, which needs to be further investigated. Overall, our findings revealed that the PDMS-2 was graded as “A”, and this scale can be used in the field of child motor development research as well as in clinical settings.

### Electronic supplementary material

Below is the link to the electronic supplementary material.

Supplementary Material 1. Table S1. COSMIN Definitions of Measurement Properties. Table S2. COSMIN Criteria for Assessing the Measurement Properties. Table S3. Levels of Evidence for the Measurement Properties of the PDMS-2.

Supplementary Material 2

## Data Availability

All the data that support the findings of this study are available from the corresponding author upon reasonable request.

## Abbreviations

COSMIN: the COnsensus based Standards for the selection of health Measurements INstruments
PDMS: 2 Peabody Developmental Motor Scales-2
EIDP: Early Intervention Developmental Profile
Bayley-III: the Bayley Scales of Infant and Toddler Development, 3rd edition
BSID-II: the Bayley Scales of Infant Development II Motor Scale
BSID-II: BOT-2 Bruininks-Oseretsky Test of Motor Proficiency-Second Edition
FUN: Miller Function and Participation Scales
ABC: Movement Assessment Battery for Children
