# Supplementary material for: Assessment of the measurement properties of the Peabody Developmental Motor Scales-2 by applying the COSMIN methodology
Source: Ital J Pediatr. 2024 Apr 24;50:87. doi: 10.1186/s13052-024-01645-6 (PMC11044420; doi:10.1186/s13052-024-01645-6)
Supplement: Supplementary file 1 — Supplementary Material 1. Table S1. COSMIN Definitions of Measurement Properties. Table S2. COSMIN Criteria for Assessing the Measurement Properties. Table S3. Levels of Evidence for the Measurement Properties of the PDMS-2. [file 13052_2024_1645_MOESM1_ESM.docx]

**Assessment of the measurement properties of the Peabody Developmental Motor Scales-2 by applying the COSMIN methodology**

Yuanye Zhu^,^ Jiahui Hu, Weibing Ye, Mallikarjuna Korivi*,Yongdong Qian*

Institute of Human Movement and Sports Engineering, College of Physical Education and Health Sciences, Zhejiang Normal University, Jinhua 321004, Zhejiang, China

E-mail: [mallik.k5@gmail.com](mailto:mallik.k5@gmail.com) ; [mallik@zjnu.edu.cn](mailto:mallik@zjnu.edu.cn)

**Table S1. COSMIN Definitions of Measurement Properties**

| Term | | | Definition |
| --- | --- | --- | --- |
| Domain | Measurement  property | Measurement  property aspect |  |
| Reliability |  |  | The degree to which the measurement is free from measurement error |
| Reliability  (extended  definition) |  |  | The extent to which scores for patients who have not changed are the same for repeated measurement under several conditions: e.g. using different sets of items from the scale (internal consistency); over time (test-retest); by different persons on the same occasion (inter-rater); or by the same persons (i.e. raters or responders) on different occasions (intra-rater) |
|  | Internal  Consistency |  | The degree of the interrelatedness among the items |
|  | Reliability |  | The proportion of the total variance in the measurements which is due to “true”a differences between patients |
|  | Measurement  Error |  | The systematic and random error of a patient’s score that is not attributed to “true”a  changes in the construct to be measured |
| Validity |  |  | The degree to which the scale measures the construct(s) it purports to measure (depressive symptom severity) |
|  | Content Validity |  | The degree to which the content of the scale is an adequate reflection of the construct to be measured |
|  |  | Face Validity | The degree to which (the items of) the scale indeed looks as though they are an adequate reflection of the construct to be measured |
|  | Construct  Validity |  | The degree to which the scores of the scale are consistent with hypotheses (for instance with regard to internal relationships, relationships to scores of other instruments, or differences between relevant groups) based on the assumption that the  PDMS-2 validly measures the construct to be measured |
|  |  | Structural Validity | The degree to which the scores of the scale are an adequate reflection of the dimensionality of the construct to be measured |
|  |  | Hypothesis Testing | Item construct validity |
|  |  | Cross-cultural  validity/measureme  nt invariance | The degree to which the performance of the items on a translated or culturally adapted scale are an adequate reflection of the performance of the items of the original version of the scale. |
| Responsiveness | Responsiveness |  | The ability of the scale to detect change over time in the construct to be measured |

**Table S2. COSMIN Criteria for Assessing the Measurement Properties**

| **Measurement**  **property** | **Rating** | **Criteria for rating** |
| --- | --- | --- |
| Structural Validity | + | Classical Test Theory: Confirmatory Factor Analysis: Comparative Fit Index or Tucker-Lewis Index or comparable measure >0.95 OR Root Mean Square Error of Approximation <0.06 or Standardized Root  Mean Residuals <0.082 |
|  | ? | Classical Test Theory: Not all information for + reported |
|  | - | Criteria for + not met |
| Internal consistency | + | At least low evidence for sufficient structural validity AND Cronbach's alpha(s) ≥ 0.70 for each unidimensional scale or subscale |
|  | ? | Criteria for “At least low evidence for sufficient structural validity” not met |
|  | - | At least low evidence for sufficient structural validity AND Cronbach’s alpha(s) < 0.70 for each unidimensional scale or subscale |
| Cross-cultural  validity/measurement  invariance | + | No important differences found between group factors (such as age, gender, language) in multiple group factor analysis OR no important differential item functioning for group factors (McFadden's R2 < 0.02) |
|  | ? | No multiple group factor analysis OR differential item functioning analysis performed |
|  | - | Important differences between group factors OR differential item functioning was found |
| Reliability | + | Intraclass correlation coefficient or weighted Kappa ≥ 0.70 |
|  | ? | Intraclass correlation coefficient or weighted Kappa not reported |
|  | - | Intraclass correlation coefficient or weighted Kappa < 0.70 |
| Measurement error | + | Smallest detectable change or limits of agreement < minimal important change |
|  | ? | Minimal important change not defined |
|  | - | Smallest Detectable Change or Limits of Agreement > Minimal Important Change |
| Criterion validity | + | Correlation with gold standard ≥ 0.70 OR Area Under the Curve ≥ 0.70 |
|  | ? | Not all information for + reported |
|  | - | Correlation with gold standard < 0.70 OR Area Under the Curve < 0.70 |
| Hypothesis testing for  construct validity | + | The result is in accordance with the hypothesis |
|  | ? | No hypothesis defined (by the review team) |
|  | - | The result is not in accordance with the hypothesis |
| Responsiveness | + | The result is in accordance with the hypothesis OR area under the curve ≥ 0.70 |
|  | ? | No hypothesis defined (by the review team) |
|  | - | The result is not in accordance with the hypothesis OR area under the curve < 0.70 |

Note:”+” = sufficient; “-” = insufficient; “?” = indeterminate

**Table S3. Levels of Evidence for the Measurement Properties of the PDMS-2**

| Measurement  properties | | Methodological risk of  bias | Inconsistency | Imprecision | Indirectness of  evidence | GRADE level  (total number of  evidence  downgrades) |
| --- | --- | --- | --- | --- | --- | --- |
| Content validity | | YES (-1)  One study of adequate methodological | NO | NO | NO | Moderate (-1) |
| Structural validity | | NO  Four studies of adequate  methodological quality | NO | NO  N> 100 | NO | High |
| Internal  consistency | | NO  Five studies of adequate  methodological quality | NO | NO  N> 100 | NO | High |
| Cross-cultural  validity | | N/A | N/A | N/A | N/A | N/A |
| Reliability | Test-retest | NO  Eight studies of adequate  methodological quality | NO | NO  N> 100 | NO | High |
|  | Inter-rater | NO  Five studies of adequate  methodological quality | NO | NO  N> 100 | NO | High |
|  | Intra-rater | NO  One study of adequate  methodological quality | NO | YES (-1)  N<100 | NO | Moderate (-1) |
| Measurement error | | NO  One study of adequate  methodological quality | NO | NO  N> 100 | NO | High |
| Hypothesis testing for construct validity | EIDP | NO  One study of adequate  methodological quality | NO | YES (-2)  N< 50 | NO | Low (-2) |
|  | M-FUN | NO  One study of adequate  methodological quality | NO | YES (-2)  N< 50 | NO | Low (-2) |
|  | Bayley-III | NO  Three studies of adequate  methodological quality | NO | NO  N> 100 | NO | High |
|  | BSID-II | NO  Three studies of adequate  methodological quality | YES (-1)  The result of 33.4% is insufficient | NO  N> 100 | NO | Moderate (-1) |
|  | BOT-2 | NO  Two studies of adequate  methodological quality | NO | NO  N> 100 | NO | High |
|  | M-ABC | NO  Two studies of adequate  methodological quality | YES (-1)  The result of 33.4% is insufficient | YES (-1)  N<100 | NO | Very low (-3) |
| Responsiveness | | YES (-2)  Two studies of inadequate  methodological quality | NO | NO  N> 100 | NO | Low (-2) |

Note: N/A = Not applicable, i.e., no included studies assessed that particular measurement property. EIDP = Early Intervention Developmental Profile, M-FUN = Miller Function and Participation Scales, Bayley-III = the Bayley Scales of Infant and Toddler Development, 3rd edition, BSID-II = the Bayley Scales of Infant Development II Motor Scale, BOT-2 = Bruininks-Oseretsky Test of Motor Proficiency-Second Edition, M-ABC = Movement Assessment Battery for Children

**Search strategy**

1.1. Search strategy for PubMed

URL: https://www.ncbi.nlm.nih.gov/pubmed/

Limits: until January 31, 2023

#1 "Peabody developmental motor scales-2" OR "PDMS-2" OR "Peabody developmental motor scales-second edition" OR “Peabody developmental motor scales-2nd”

#2 (instrumentation[sh] OR methods[sh] OR “Validation Studies”[pt] OR “Comparative Study”[pt] OR “psychometrics”[MeSH] OR psychometr*[tiab] OR clinimetr*[tw] OR clinometr*[tw] OR “outcome assessment (health care)”[MeSH] OR “outcome assessment”[tiab] OR “outcome measure*”[tw] OR “observer variation”[MeSH] OR “observer variation”[tiab] OR “Health Status Indicators”[Mesh] OR “reproducibility of results”[MeSH] OR reproducib*[tiab] OR “discriminant analysis”[MeSH] OR reliab*[tiab] OR unreliab*[tiab] OR valid*[tiab] OR “coefficient of variation”[tiab] OR coefficient[tiab] OR homogeneity[tiab] OR homogeneous[tiab] OR “internal consistency”[tiab] OR (cronbach*[tiab] AND (alpha[tiab] OR alphas[tiab])) OR (item[tiab] AND (correlation*[tiab] OR selection*[tiab] OR reduction*[tiab])) OR agreement[tw] OR precision[tw] OR imprecision[tw] OR “precise values”[tw] OR test-retest[tiab] OR (test[tiab] AND retest[tiab]) OR (reliab*[tiab] AND (test[tiab] OR retest[tiab])) OR stability[tiab] OR interrater[tiab] OR inter-rater[tiab] OR intrarater[tiab] OR intra-rater[tiab] OR intertester[tiab] OR inter-tester[tiab] OR intratester[tiab] OR intra-tester[tiab] OR interobserver[tiab] OR inter-observer[tiab] OR intraobserver[tiab] OR intra-observer[tiab] OR intertechnician[tiab] OR inter-technician[tiab] OR intratechnician[tiab] OR intra-technician[tiab] OR interexaminer[tiab] OR inter-examiner[tiab] OR intraexaminer[tiab] OR intra-examiner[tiab] OR interassay[tiab] OR inter-assay[tiab] OR intraassay[tiab] OR intra-assay[tiab] OR interindividual[tiab] OR inter-individual[tiab] OR intraindividual[tiab] OR intra-individual[tiab] OR interparticipant[tiab] OR inter-participant[tiab] OR intraparticipant[tiab] OR intra-participant[tiab] OR kappa[tiab] OR kappa’s[tiab] OR kappas[tiab] OR repeatab*[tw] OR ((replicab*[tw] OR repeated[tw]) AND (measure[tw] OR measures[tw] OR findings[tw] OR result[tw] OR results[tw] OR test[tw] OR tests[tw])) OR generaliza*[tiab] OR generalisa*[tiab] OR concordance[tiab] OR (intraclass[tiab] AND correlation*[tiab]) OR discriminative[tiab] OR “known group”[tiab] OR “factor analysis”[tiab] OR “factor analyses”[tiab] OR “factor structure”[tiab] OR “factor structures”[tiab] OR dimension*[tiab] OR subscale*[tiab] OR (multitrait[tiab] AND scaling[tiab] AND (analysis[tiab] OR analyses[tiab])) OR “item discriminant”[tiab] OR “interscale correlation*”[tiab] OR error[tiab] OR errors[tiab] OR “individual variability”[tiab] OR “interval variability”[tiab] OR “rate variability”[tiab] OR (variability[tiab] AND (analysis[tiab] OR values[tiab])) OR (uncertainty[tiab] AND (measurement[tiab] OR measuring[tiab])) OR “standard error of measurement”[tiab] OR sensitiv*[tiab] OR responsive*[tiab] OR (limit[tiab] AND detection[tiab]) OR “minimal detectable concentration”[tiab] OR interpretab*[tiab] OR ((minimal[tiab] OR minimally[tiab] OR clinical[tiab] OR clinically[tiab]) AND (important[tiab] OR significant[tiab] OR detectable[tiab]) AND (change[tiab] OR difference[tiab])) OR (small*[tiab] AND (real[tiab] OR detectable[tiab]) AND (change[tiab] OR difference[tiab])) OR “meaningful change”[tiab] OR “ceiling effect”[tiab] OR “floor effect”[tiab] OR “Item response model”[tiab] OR IRT[tiab] OR Rasch[tiab] OR “Differential item functioning”[tiab] OR DIF[tiab] OR “computer adaptive testing”[tiab] OR “item bank”[tiab] OR “cross-cultural equivalence”[tiab])

#3 (“addresses”[Publication Type] OR “biography”[Publication Type] OR “case reports”[Publication Type] OR “comment”[Publication Type] OR “directory”[Publication Type] OR “editorial”[Publication Type] OR “festschrift”[Publication Type] OR “interview”[Publication Type] OR “lectures”[Publication Type] OR “legal cases”[Publication Type] OR “legislation”[Publication Type] OR “letter”[Publication Type] OR “news”[Publication Type] OR “newspaper article”[Publication Type] OR “patient education handout”[Publication Type] OR “popular works”[Publication Type] OR “congresses”[Publication Type] OR “consensus development conference”[Publication Type] OR “consensus development conference, nih”[Publication Type] OR “practice guideline”[Publication Type]) NOT (“animals”[MeSH Terms] NOT “humans”[MeSH Terms])

#4 #1 AND #2 NOT #3

1.2. Search strategy for CINAHL

URL: https://search.ebscohost.com

Limits: until January 31, 2023

#1 SU "Peabody developmental motor scales-2" OR "PDMS-2" OR "Peabody developmental motor scales-second edition" OR “Peabody developmental motor scales-2nd”

#2 TI psychometr* OR TI observer variation OR TI reproducib* OR TI reliab* OR TI unreliab* OR TI valid* OR TI coefficient OR TI homogeneity OR TI homogeneous OR TI “internal consistency” OR AB psychometr* OR AB observer variation OR AB reproducib* OR AB reliab* OR AB unreliab* OR AB valid* OR AB coefficient OR AB homogeneity OR AB homogeneous OR AB “internal consistency” OR (TI cronbach* OR AB cronbach* AND (TI alpha OR AB alpha OR TI alphas OR AB alphas)) OR (TI item OR AB item AND (TI correlation* OR AB correlation* OR TI selection* OR AB selection* OR TI reduction* OR AB reduction*)) OR TI agreement OR TI precision OR TI imprecision OR TI “precise values” OR TI test-retest OR AB agreement OR AB precision OR AB imprecision OR AB “precise values” OR AB test-retest OR (TI test OR AB test AND TI retest OR AB retest) OR (TI reliab* OR AB reliab* AND (TI test OR AB test OR TI retest or AB retest)) OR TI stability OR TI interrater OR TI interrater OR TI intrarater OR TI intra-rater OR TI intertester OR TI inter-tester OR TI intratester OR TI intra-tester OR TI interobserver OR TI inter-observer OR TI intraobserver OR TI intra-observer OR TI intertechnician OR TI inter-technician OR TI intratechnician OR TI intra-technician OR TI interexaminer OR TI inter-examiner OR TI intraexaminer OR TI intra-examiner OR TI interassay OR TI inter-assay OR TI intraassay OR TI intra-assay OR TI interindividual OR TI inter-individual OR TI intraindividual OR TI intra-individual OR TI interparticipant OR TI inter-participant OR TI intraparticipant OR TI intra-participant OR TI kappa OR TI kappa’s OR TI kappas OR TI repeatab* OR AB stability OR AB interrater OR AB inter-rater OR AB intrarater OR AB intra-rater OR AB intertester OR AB inter-tester OR AB intratester OR AB intra-tester OR AB interobserver OR AB inter-observer OR AB intraobserver OR AB intra-observer OR AB intertechnician OR AB inter-technician OR AB intratechnician OR AB intra-technician OR AB interexaminer OR AB inter-examiner OR AB intraexaminer OR AB intra-examiner OR AB interassay OR AB inter-assay OR AB intraassay OR AB intra-assay OR AB interindividual OR AB inter-individual OR AB intraindividual OR AB intra-individual OR AB interparticipant OR AB inter-participant OR AB intraparticipant OR AB intra-participant OR AB kappa OR AB kappa’s OR AB kappas OR AB repeatab* OR ((TI replicab* OR AB replicab* OR TI repeated OR AB repeated) AND (TI measure OR AB measure OR TI measures OR AB measures OR TI findings OR AB findings OR TI result OR AB result OR TI results OR AB results OR TI test OR AB test OR TI tests OR AB tests)) OR TI generaliza* OR TI generalisa* OR TI concordance OR AB generaliza* OR AB generalisa* OR AB concordance OR (TI intraclass OR AB intraclass AND TI correlation* or AB correlation*) OR TI discriminative OR TI “known group” OR TI factor analysis OR TI factor analyses OR TI dimension* OR TI subscale* OR AB discriminative OR AB “known group” OR AB factor analysis OR AB factor analyses OR AB dimension* OR AB subscale* OR (TI multitrait OR AB multitrait AND TI scaling OR AB scaling AND (TI analysis OR AB analysis OR TI analyses OR AB analyses)) OR TI item discriminant OR TI interscale correlation* OR TI error OR TI errors OR TI “individual variability” OR AB item discriminant OR AB interscale correlation* OR AB error OR AB errors OR AB “individual variability” OR (TI variability OR AB variability AND (TI analysis OR AB analysis OR TI values OR AB values)) OR (TI uncertainty OR AB uncertainty AND (TI measurement OR AB measurement OR TI measuring OR AB measuring)) OR TI “standard error of measurement” OR TI sensitiv* OR TI responsive* OR AB “standard error of measurement” OR AB sensitiv* OR AB responsive* OR ((TI minimal OR TI minimally OR TI clinical OR TI clinically OR AB minimal OR AB minimally OR AB clinical OR AB clinically) AND (TI important OR TI significant OR TI detectable OR AB important OR AB significant OR AB detectable) AND (TI change OR AB change OR TI difference OR AB difference)) OR (TI small* OR AB small* AND (TI real OR AB real OR TI detectable OR AB detectable) AND (TI change OR AB change OR TI difference OR AB difference)) OR TI meaningful change OR TI “ceiling effect” OR TI “floor effect” OR TI “Item response model” OR TI IRT OR TI Rasch OR TI “Differential item functioning” OR TI DIF OR TI “computer adaptive testing” OR TI “item bank” OR TI “cross-cultural equivalence” OR TI outcome assessment OR AB meaningful change OR AB “ceiling effect” OR AB “floor effect” OR AB “Item response model” OR AB IRT OR AB Rasch OR AB “Differential item functioning” OR AB DIF OR AB “computer adaptive testing” OR AB “item bank” OR AB “cross-cultural equivalence” OR AB outcome assessment

#3  (address OR biography OR “case reports” OR comment OR directory OR editorial OR festschrift OR interview OR lecture OR “legal case” OR legislation OR letter OR news OR “newspaper article” OR “patient education handout” OR “popular work” OR congress OR “consensus development conference” OR “consensus development conference, NIH” OR “practice guideline”).mp

#4 #1 AND #2 NOT #3

1.3. Search strategy for Web of science AND MEDLINE

URL: https://www.webofscience.com

Limits: until January 31, 2023

#1 (TS=("Peabody developmental motor scales-2" OR "PDMS-2" OR "Peabody developmental motor scales-second edition" OR “Peabody developmental motor scales-2nd” ))

#2 (TS=(instrumentation OR methods) OR TS=(“validation study” OR “comparative study”) OR TS=(Psychometrics) OR (TI=(psychometr*)) OR AB=(psychometr*) OR TS=(clinimetr* OR clinometr*) OR TS=(“Outcome Assessment, Health Care”) OR (TI=(“outcome assessment”)) OR AB=(“outcome assessment”) OR TS=(“outcome measure*”) OR TS=(“Observer Variation”) OR TI=(“observer variation”) OR AB=(“observer variation”) OR TS=(“Health Status Indicators”) OR TS=(“Reproducibility of Results”) OR (TI=(reproducib*)) OR AB=(reproducib*) OR TS=(“Discriminant Analysis”) OR (TI=((reliab* OR unreliab* OR valid* OR “coefficient of variation” OR coefficient OR homogeneity OR homogeneous OR “internal consistency”))) OR AB=((reliab* OR unreliab* OR valid* OR “coefficient of variation” OR coefficient OR homogeneity OR homogeneous OR “internal consistency”)) OR (TI=(( cronbach* AND (alpha OR alphas)))) OR AB=(( cronbach* AND (alpha OR alphas))) OR (TI=((item AND (correlation* OR selection* OR reduction*)))) OR AB=((item AND (correlation* OR selection* OR reduction*))) OR TS=(agreement) OR TS=(precision) OR TS=(imprecision) OR TS=(“precise values”) OR (TI=(test-retest)) OR AB=(test-retest) OR (TI=((test AND retest))) OR AB=((test AND retest)) OR (TI=((reliab* AND (test OR retest)))) OR AB=((reliab* AND (test OR retest))) OR (TI=(stability)) OR AB=(stability) OR (TI=((interrater OR inter-rater OR intrarater OR intra-rater))) OR AB=((interrater OR inter-rater OR intrarater OR intra-rater)) OR (TI=((intertester OR inter-tester OR intratester OR intra-tester))) OR AB=((intertester OR inter-tester OR intratester OR intra-tester)) OR (TI=((interobserver OR inter-observer OR intraobserver OR intra-observer))) OR AB=((interobserver OR inter-observer OR intraobserver OR intra-observer)) OR (TI=((intertechnician OR inter-technician OR intratechnician OR intra-technician))) OR AB=((intertechnician OR inter-technician OR intratechnician OR intra-technician)) OR (TI=((interexaminer OR inter-examiner OR intraexaminer OR intra-examiner))) OR AB=((interexaminer OR inter-examiner OR intraexaminer OR intra-examiner)) OR (TI=((interassay OR inter-assay OR intraassay OR intra-assay))) OR AB=((interassay OR inter-assay OR intraassay OR intra-assay)) OR (TI=((interindividual OR inter-individual OR intraindividual OR intra-individual))) OR AB=((interindividual OR inter-individual OR intraindividual OR intra-individual)) OR (TI=((interparticipant OR inter-participant OR intraparticipant OR intra-participant))) OR AB=((interparticipant OR inter-participant OR intraparticipant OR intra-participant)) OR (TI=(kappa)) OR AB=(kappa) OR (TI=(kappas)) OR AB=(kappas) OR TS=(repeatab*) OR TS=(((replicab* OR repeated) AND (measure OR measures OR findings OR result OR results OR test OR tests))) OR (TI=((generaliza* OR generalisa*))) OR AB=((generaliza* OR generalisa*)) OR (TI=(concordance)) OR AB=(concordance) OR (TI=((intraclass AND correlation*))) OR AB=((intraclass AND correlation*)) OR (TI=(discriminative)) OR AB=(discriminative) OR (TI=(“known group”)) OR AB=(“known group”) OR (TI=((“factor analysis” OR “factor analyses” OR “factor structure” OR “factor structures”))) OR AB=((“factor analysis” OR “factor analyses” OR “factor structure” OR “factor structures”)) OR (TI=(dimension*)) OR AB=(dimension*) OR (TI=(subscale*)) OR AB=(subscale*) OR (TI=((multitrait AND scaling AND (analysis OR analyses)))) OR AB=((multitrait AND scaling AND (analysis OR analyses))) OR (TI=(“item discriminant”)) OR AB=(“item discriminant”) OR (TI=(“interscale correlation*”)) OR AB=(“interscale correlation*”) OR (TI=((error OR errors))) OR AB=((error OR errors)) OR (TI=(“individual variability”)) OR AB=(“individual variability”) OR (TI=(“interval variability”)) OR AB=(“interval variability”) OR (TI=(“rate variability”)) OR AB=(“rate variability”) OR (TI=((variability AND (analysis OR values)))) OR AB=((variability AND (analysis OR values))) OR (TI=((uncertainty AND (measurement OR measuring)))) OR AB=((uncertainty AND (measurement OR measuring))) OR (TI=(“standard error of measurement”)) OR AB=(“standard error of measurement”) OR (TI=(sensitiv*)) OR AB=(sensitiv*) OR (TI=(responsive*)) OR AB=(responsive*) OR (TI=((limit AND detection))) OR AB=((limit AND detection)) OR (TI=(“minimal detectable concentration”)) OR AB=(“minimal detectable concentration”) OR (TI=(interpretab*)) OR AB=(interpretab*) OR (TI=(((minimal OR minimally OR clinical OR clinically) AND (important OR significant OR detectable) AND (change OR difference)))) OR AB=(((minimal OR minimally OR clinical OR clinically) AND (important OR significant OR detectable) AND (change OR difference))) OR (TI=((small* AND (real OR detectable) AND (change OR difference)))) OR AB=((small* AND (real OR detectable) AND (change OR difference))) OR (TI=(“meaningful change”)) OR AB=(“meaningful change”) OR (TI=(“ceiling effect”)) OR AB=(“ceiling effect”) OR (TI=(“floor effect”)) OR AB=(“floor effect”) OR (TI=(“item response model”)) OR AB=(“item response model”) OR (TI=(IRT)) OR AB=(IRT) OR (TI=(rasch)) OR AB=(rasch) OR (TI=(“differential item functioning”)) OR AB=(“differential item functioning”) OR (TI=(DIF)) OR AB=(DIF) OR (TI=(“computer adaptive testing”)) OR AB=(“computer adaptive testing”) OR (TI=(“item bank”)) OR AB=(“item bank”) OR (TI=(“cross-cultural equivalence”)) OR AB=(“cross-cultural equivalence”))

#3 (TS=((address OR biography OR “case reports” OR comment OR directory OR editorial OR festschrift OR interview OR lecture OR “legal case” OR legislation OR letter OR news OR “newspaper article” OR “patient education handout” OR “popular work” OR congress OR “consensus development conference” OR “consensus development conference, NIH” OR “practice guideline”)))

#4 #1 AND #2 NOT #3

1.4. Search strategy for EMbase

URL: https://www.embase.com

Limits: until January 31, 2023

#1 (‘Peabody developmental motor scales-2’ OR ‘PDMS-2’ OR ‘Peabody developmental motor scales-second edition’ OR ‘Peabody developmental motor scales-2nd’)

#2 ‘intermethod comparison’/exp OR ‘data collection method’/exp OR ‘validation study’/exp OR ‘feasibility study’/exp OR ‘pilot study’/exp OR ‘psychometry’/exp OR ‘reproducibility’/exp OR reproducib*:ab,ti OR ‘audit’:ab,ti OR psychometr*:ab,ti OR clinimetr*:ab,ti OR clinometr*:ab,ti OR ‘observer variation’/exp OR ‘observer variation’:ab,ti OR ‘discriminant analysis’/exp OR ‘validity’/exp OR reliab*:ab,ti OR valid*:ab,ti OR‘coefficient’:ab,ti OR ‘internal consistency’:ab,ti OR (cronbach*:ab,ti AND (‘alpha’:ab,ti OR ‘alphas’:ab,ti)) OR ‘item correlation’:ab,ti OR ‘item correlations’:ab,ti OR ‘item selection’:ab,ti OR ‘item selections’:ab,ti OR ‘item reduction’:ab,ti OR ‘item reductions’:ab,ti OR ‘agreement’:ab,ti OR ‘precision’:ab,ti OR ‘imprecision’:ab,ti OR ‘precise values’:ab,ti OR ‘test-retest’:ab,ti OR (‘test’:ab,ti AND ‘retest’:ab,ti) OR (reliab*:ab,ti AND (‘test’:ab,ti

OR ‘retest’:ab,ti)) OR ‘stability’:ab,ti OR ‘interrater’:ab,ti OR ‘inter-rater’:ab,ti OR ‘intrarater’:ab,ti OR ‘intra-rater’:ab,ti OR ‘intertester’:ab,ti OR ‘inter-tester’:ab,ti OR ‘intratester’:ab,ti OR ‘intratester’:ab,ti OR ‘interobeserver’:ab,ti OR ‘inter-observer’:ab,ti OR ‘intraobserver’:ab,ti OR ‘intraobserver’:ab,ti OR ‘intertechnician’:ab,ti OR ‘inter-technician’:ab,ti OR ‘intratechnician’:ab,ti OR ‘intratechnician’:ab,ti

OR ‘interexaminer’:ab,ti OR ‘inter-examiner’:ab,ti OR ‘intraexaminer’:ab,ti OR‘intraexaminer’:ab,ti OR ‘interassay’:ab,ti OR ‘inter-assay’:ab,ti OR ‘intraassay’:ab,ti OR ‘intra-assay’:ab,ti OR ‘interindividual’:ab,ti OR ‘inter-individual’:ab,ti OR ‘intraindividual’:ab,ti OR ‘intra-individual’:ab,ti OR ‘interparticipant’:ab,ti OR ‘inter-participant’:ab,ti OR ‘intraparticipant’:ab,ti OR ‘intraparticipant’:ab,ti OR ‘kappa’:ab,ti OR ‘kappas’:ab,ti OR ‘coefficient of variation’:ab,ti OR repeatab*:ab,ti OR (replicab*:ab,ti OR ‘repeated’:ab,ti AND (‘measure’:ab,ti OR ‘measures’:ab,ti OR ‘findings’:ab,ti OR ‘result’:ab,ti OR ‘results’:ab,ti OR ‘test’:ab,ti OR ‘tests’:ab,ti)) OR generaliza*:ab,ti OR generalisa*:ab,ti OR ‘concordance’:ab,ti OR (‘intraclass’:ab,ti AND correlation*:ab,ti) OR ‘discriminative’:ab,ti OR ‘known group’:ab,ti OR ‘factor analysis’:ab,ti OR ‘factor analyses’:ab,ti OR ‘factor structure’:ab,ti OR ‘factor structures’:ab,ti OR ‘dimensionality’:ab,ti OR subscale*:ab,ti OR

‘multitrait scaling analysis’:ab,ti OR ‘multitrait scaling analyses’:ab,ti OR ‘item discriminant’:ab,ti OR ‘interscale correlation’:ab,ti OR ‘interscale correlations’:ab,ti OR (‘error’:ab,ti OR ‘errors’:ab,ti AND (measure*:ab,ti OR correlat*:ab,ti OR evaluat*:ab,ti OR ‘accuracy’:ab,ti OR ‘accurate’:ab,ti OR ‘precision’:ab,ti OR ‘mean’:ab,ti)) OR ‘individual variability’:ab,ti OR ‘interval variability’:ab,ti OR ‘rate variability’:ab,ti OR ‘variability analysis’:ab,ti OR (‘uncertainty’:ab,ti AND (‘measurement’:ab,ti OR ‘measuring’:ab,ti)) OR ‘standard error of measurement’:ab,ti OR sensitiv*:ab,ti OR responsive*:ab,ti OR (‘limit’:ab,ti AND ‘detection’:ab,ti) OR ‘minimal detectable concentration’:ab,ti OR interpretab*:ab,ti OR (small*:ab,ti AND (‘real’:ab,ti OR ‘detectable’:ab,ti) AND (‘change’:ab,ti OR ‘difference’:ab,ti)) OR ‘meaningful change’:ab,ti OR ‘minimal important change’:ab,ti OR ‘minimal important difference’:ab,ti OR ‘minimally important change’:ab,ti OR ‘minimally important difference’:ab,ti OR ‘minimal detectable change’:ab,ti OR ‘minimal detectable difference’:ab,ti OR ‘minimally detectable change’:ab,ti OR ‘minimally detectable difference’:ab,ti OR ‘minimal real change’:ab,ti OR ‘minimal real difference’:ab,ti OR ‘minimally real change’:ab,ti OR ‘minimally real difference’:ab,ti OR ‘ceiling effect’:ab,ti OR ‘floor effect’:ab,ti OR ‘item response model’:ab,ti OR ‘irt’:ab,ti OR ‘rasch’:ab,ti OR ‘differential item functioning’:ab,ti OR ‘dif’:ab,ti OR ‘computer adaptive testing’:ab,ti OR ‘item bank’:ab,ti OR ‘cross-cultural equivalence’:ab,ti)

#3 #1 AND #2
