# Supplementary material for: Assessment of the measurement properties of the Peabody Developmental Motor Scales-2 by applying the COSMIN methodology
Source: Ital J Pediatr. 2024 Apr 24;50:87. doi: 10.1186/s13052-024-01645-6 (PMC11044420; doi:10.1186/s13052-024-01645-6)
Supplement: Supplementary file 2 — Supplementary Material 2 [file 13052_2024_1645_MOESM2_ESM.docx]

|  | Institute of Human Movement and Sports Engineering |
| --- | --- |
|  | College of Physical Education and Health Sciences, Zhejiang Normal University  688 Yingbin Avenue, Jinhua, Zhejiang 321004, China |

 To	**05 Jan 2024**

**Prof. Raffaele Falsaperla, Ph. D., M.D.**

Italian Journal of Pediatrics

Dear **Prof. Raffaele**, Greetings!!!

We are highly thankful to you for giving this opportunity to revise our manuscript. We appreciate Reviewers’ efforts for their careful evaluation and valuable comments on our manuscript.

According to your direction and Reviewers’ comments, we have addressed all the comments, and revised our manuscript carefully. All corrections we have done in the manuscript and were marked in ‘red’ color for better understanding. We also provided our point-by-point responses to each comment of the Reviewers. The revised version of the manuscript has been edited by AJE for language corrections.

We do believe that the changes we have done in our manuscript have improved the overall quality. The revised version is suitable for your kind perusal. Once again thank you very much for considering our manuscript in ‘Italian Journal of Pediatrics’.

We are looking forward to hearing the good news from you

Sincerely yours,

Prof. Mallikarjuna Korivi, Ph.D.

College of PE and Health Sciences

Zhejiang Normal University

688 Yingbin Avenue, Jinhua City, Zhejiang Province 321004, China

E-mail: [**mallik.k5@gmail.com**](mailto:mallik.k5@gmail.com) **/** [**mallik@zjnu.edu.cn**](mailto:mallik@zjnu.edu.cn)

Phone: +86-183-29032055
